# Targeted microbubbles combined with low-power focused ultrasound promote the thrombolysis of acute deep vein thrombosis

**DOI:** 10.3389/fbioe.2023.1163405

**Published:** 2023-03-16

**Authors:** Jianfu Chen, Yuan Yang, Yunyan Li, Lirong Xu, Chun Zhao, Qi Chen, Yongping Lu

**Affiliations:** ^1^ Kunming Medical University, Kunming, Yunnan, China; ^2^ Department of Ultrasound, The Affiliated Hospital of Yunnan University (The Second People’s Hospital of Yunnan Province), Kunming, Yunnan, China; ^3^ School of Clinical Medicine, Dali University, Dali, Yunnan, China

**Keywords:** targeted microbubbles, low-power focused ultrasound, ultrasonic irradiation, acute deep vein thrombosis, thrombolysis

## Abstract

**Introduction:** The side effects of conventional therapy for acute deep vein thrombosis (DVT) are severe, with inflammatory reactions playing a pivotal role. It is particularly important to explore new ways of treatment thrombosis by targeting inflammatory factors.

**Methods:** A targeted microbubble contrast agent was prepared using the biotin-avidin method. The 40 DVT model rabbits were established and divided into four groups according to different treatment regimens. The four coagulation indexes, TNF-α, and D-dimer content of experimental animals were measured before modeling and before and after treatment, and the thrombolysis was assessed by ultrasound imaging. Finally, the results were verified by pathology.

**Results and Discussion:** Fluorescence microscopy verified the successful preparation of targeted microbubbles. Among the groups, PT, APTT, and TT in Group II-IV were longer than those in Group I (all *p* < 0.05). FIB and D-dimer content were lower than those in Group I (all *p* < 0.05), and TNF-α content in Group IV was lower than that in Group I-III (all *p* < 0.05). Pairwise comparison before modeling and before treatment and after treatment showed that, after treatment, the PT, APTT, and TT in Group II-IV were longer than those before modeling (all *p* < 0.05). The contents of FIB and D-dimer were lower than those before modeling and before treatment (all *p* < 0.05). The content of TNF-α decreased significantly only in Group IV, but increased in the other three groups. Targeted microbubbles combined with Low-power focused ultrasound can reduce inflammation, significantly promote thrombolysis, and provide new ideas and methods for the diagnosis and treatment of acute DVT.

## 1 Introduction

Venous thromboembolism has been ranked as the third most common cause of disability and cardiovascular death in the world, encompassing deep vein thrombosis (DVT) and pulmonary embolism (PE), which frequently occur due to trauma, stress, pregnancy, and postoperative complications (Khan et al., 2021; Jackson et al., 2022). Due to the intensive study of thrombosis, venous thrombosis has been revealed be closely associated with the inflammatory response of the venous wall. Slowed blood flow, hypoxia, inflammatory cell activation, and the release of various active substances correlated with thrombosis (Poredos and Poredos, 2022). Studies have revealed that TNF-α is closely linked to the interaction between leukocytes and endothelial cells (Aguilar et al., 2021). In the initial stage of thrombosis, TNF-α induces the expression of adhesion molecules on the surface of endothelial cells. Therefore, the inflammatory response can be neutralized by inhibiting TNF-α. The use of antibody to TNF-α is one of the most effective methods to reduce neutrophil infiltration (Presicce et al., 2020). Aue et al. (2011) found that the upregulation of TNF-α was significantly related to thrombosis, and the upregulation degree was more predictive than the medical history of non-drug-related (DVT).

The above descriptions indicate that TNF-α can be used as a target for thrombosis therapy. Anti-inflammatory and targeted therapy of thrombosis can be achieved by using bioactive materials (Liu et al., 2022a) or ultrasound microbubble contrast agents labeled with targeting guides. The combination of a microbubble contrast agent and ultrasound targeted microbubble destruction (UTMD) technology can deliver bioactive substances to various targets non-invasively and specifically (Liu et al., 2022b). At present, it is mainly used in the treatment of heart disease, tumors, and atherosclerotic plaque (Zhang et al., 2021; He et al., 2022). UTMD technology breaks up targeted microbubbles and releases large amounts of therapeutic substances in the target area, which optimizes the local treatment effect while reducing systemic side effects (Yang et al., 2019; Xu et al., 2022).

There is no doubt that ultrasound can accelerate thrombolysis. Both high-frequency ultrasound and low-power ultrasound can significantly promote thrombolysis (Notten et al., 2020; Avgerinos et al., 2021). Microbubbles combined with thrombolytic medication, such as urokinase (UK) or tissue plasminogen activator, can further enhance the success rate of thrombolysis (Wang et al., 2019; Wang et al., 2020). Studies have demonstrated that UTMD can mechanically remove dialysis grafts with thrombosis in experimental animals even without the delivery of any substance (Liang et al., 2021). Ultrasound with a frequency greater than 0.5 MHz can enhance the dissolution of thrombus *in vitro* (Ebben et al., 2015), and the study by Zhong et al. (2019) has confirmed that ultrasound can accelerate thrombolysis *in vivo*. In recent years, microbubbles carrying medication to treat thrombosis have also become a research hotspot.

Microbubbles carrying the antibody of TNF-α played multiple roles in thrombolytic therapy. Firstly, targeted microbubbles were an ultrasound contrast agent, which could effectively identify thrombus through ultrasound contrast imaging and evaluate the thrombolytic effect. Secondly, the monoclonal antibody to TNF-α carried by targeted microbubbles bound the local TNF-α produced by thrombus and alleviated the inflammatory response. Finally, the cavitation effect and thermal effect generated by breaking the microbubble through UTMD technology stimulated the regeneration of blood vessels, loosened the internal structure of thrombus, and promoted the effect of thrombolytic drugs.

In this study, an animal model of DVT was established and treated by low-power ultrasound combined with targeted microbubbles (MBt) bearing TNF-α antibody and UK. UTMD technology promoted monoclonal antibodies to TNF-α release from MBt and alleviated inflammation and subsequent chain reaction. At the same time, the cavitation effect produced by ultrasonic crushing microbubbles also promoted thrombus dissolution, in the hope of achieving the effect of complete dissolution of thrombus, recanalization, and providing new ideas for thrombus treatment.

## 2 Materials and methods

### 2.1 Establishment of thrombus model

In this experiment, 40 healthy adult Japanese white rabbits were used to make the acute DVT model. The experimental animals were supplemented according to the principle of randomization, if some of them died during the experiment.

Firstly, the rabbit inferior vena cava was exposed and dissociated. The dissociative segment was from the lower part of the renal vein bifurcation to the upper part of the iliac vein bifurcation. Thread was threaded under the renal vein bifurcation. The self-made needle tube was positioned parallel to the inferior vena cava. The vena cava and needle tube were ligated with a suture. After ligation, the needle tube was removed to ensure the identical stenosis rate of each rabbit. The distal blood vessel was clamped with a vascular clamp and removed 30 min later, at which point the abdomen was sutured. Finally, Philips Ultrasonic diagnostic instrument (EPIQ 5, Holland) was used to observe and confirm the inferior vena cava thrombosis.

### 2.2 Reagents

#### 2.2.1 UK

UK (100,000 IU/bottle, Wuhan Renfu Pharmaceutical Co., Ltd.) was dissolved and diluted with normal saline, and slowly injected through the rabbit ear vein at a proportion of 10,000 IU/kg.

#### 2.2.2 Targeted microbubble contrast agent

USphere^™^ Labeler microbubble (particle size: 492.66 nm ± 41.68 nm, marked avidin, Taiwan, China Boxin Biotechnology Co., Ltd.) was placed in the room for 30 min. After it returned to room temperature, 100 uL monoclonal antibody of TNF-α (marked biotin and FAM fluorescent group, Nanjing Zhongding Biotechnology Co., Ltd.) was injected into it. The mixture was oscillated for 40 s in an oscillator until it became white microbubbles.

### 2.3 Grouping and treatment of experimental animals

#### 2.3.1 Grouping

There were 40 rabbits randomly divided into four groups, with 10 rabbits in each group. Group I: ultrasonic irradiation; Group II: UK + ultrasonic irradiation; Group III: UK + control microbubbles (MBc) + ultrasonic irradiation; Group IV: UK + MBt + ultrasonic irradiation.

The treatment time of the above four groups was 3 days. After the final treatment, the experimental animals were killed and the thrombus tissue was taken.

#### 2.3.2 Treatment method

Ultrasonic irradiation was performed using a Low-Power Focused Ultrasound Device (Chongqing Ronghai Ultrasonic Medical Engineering Co., Ltd.). The frequency was 0.7 MHz, the power was 2.0 w/cm^2^, and the vascular lumen of thrombus segment was irradiated continuously for 20 min. UK’s dose was 10,000 IU/kg; MBc’s dose: 0.05 mL/kg; MBt’s dose: 0.05 mL/kg, and all reagents were injected through the auricular vein of rabbits.

### 2.4 Evaluation of DVT

After the final treatment, DVT was detected and evaluated by color Doppler flow imaging (CDFI) and Contrast-Enhanced Ultrasound (CEUS), and the patency of the inferior vena cava was analyzed.

### 2.5 Laboratory index

The thrombin time (TT), prothrombin time (PT), activated partial thrombin time (APTT), and fibrinogen (FIB) in peripheral blood of experimental animals was measured before modelling, before treatment, and after treatment respectively. The content of D-dimer and TNF-α was detected by enzyme-linked immunosorbent assay (ELISA).

### 2.6 Pathological examination

IVC thrombus was removed, fixed with 4% paraformaldehyde, and embedded in paraffin. Hematoxylin-eosin staining was used to observe the morphology of the thrombus.

### 2.7 Statistical analyses

The experimental data were analyzed using SPSS 23.0 statistical software. The measurement data were expressed as mean ± standard deviation. Multiple samples were compared by one-way ANOVA, pairwise comparison by LSD-*t* test. The difference was considered statistically significant at *p* < 0.05.

## 3 Result

### 3.1 Identification of targeted microbubble contrast agent

**FIGURE 1:**
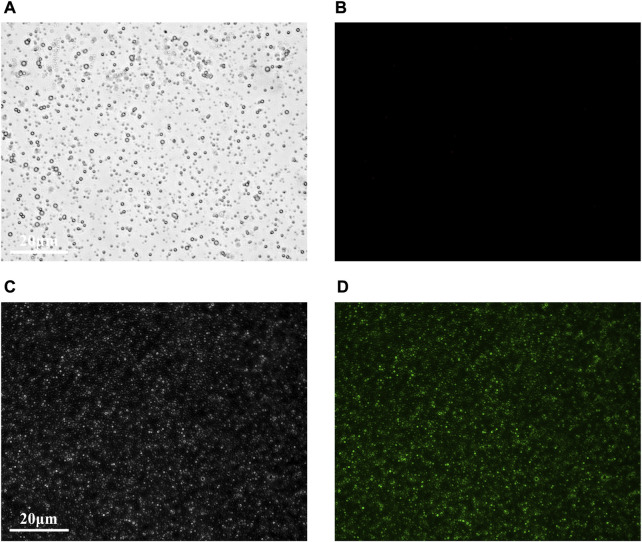
Fluorescence microscopic images of control microbubbles **(A,B)** and targeted microbubbles **(C,D)**. **(A,C)** were bright field images and **(B,D)** were FAM fluorescence images. Scale bar = 20 μm.

Under the light microscope, the MBc and MBt were regular in shape, uniform in size and distribution (Figures 1A, C), and there was no obvious morphological difference between them. The MBc had no fluorescence imaging under the inverted fluorescence interference microscope (Figure 1B), while the MBt showed obvious green fluorescence (Figure 1D), indicating that the TNF-α antibody was successfully coupled with USphere^™^ Labeler microbubble contrast agent.

### 3.2 Evaluation of ultrasonic imaging

#### 3.2.1 Evaluation of DVT by CDFI

**FIGURE 2:**
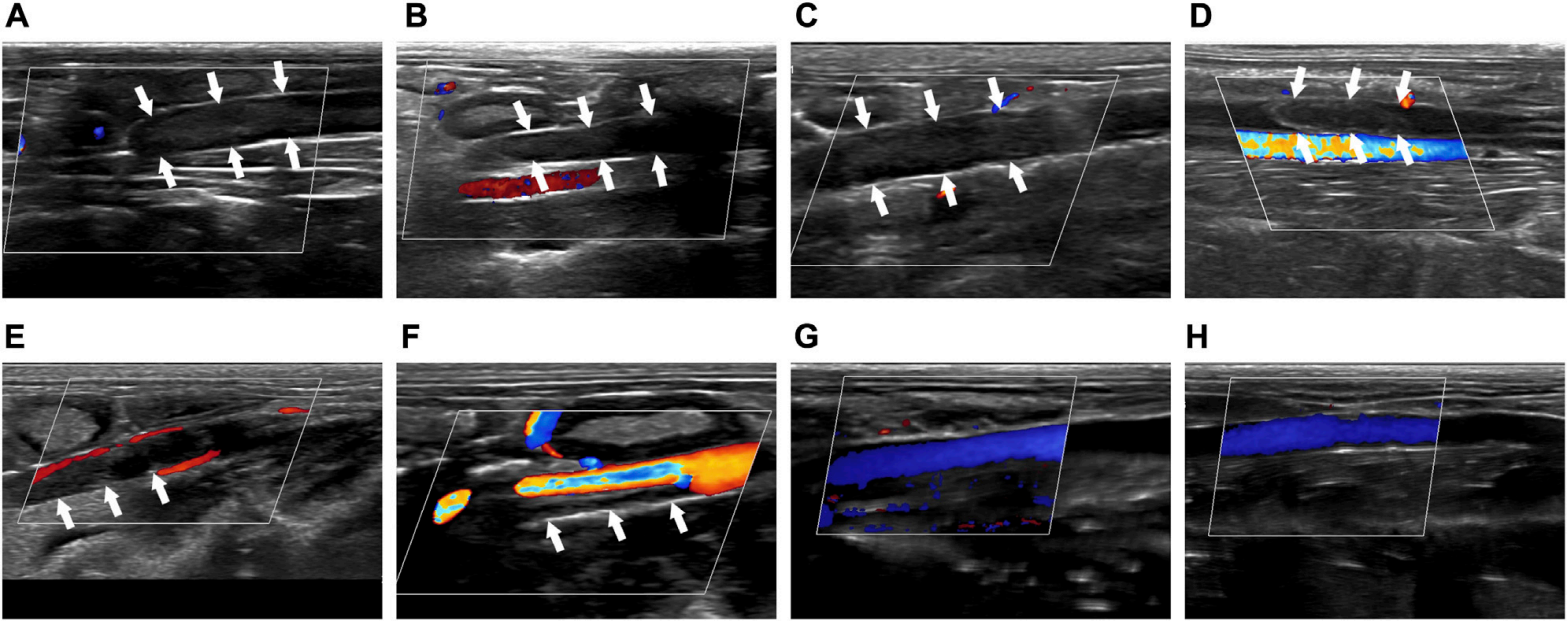
Comparison of imaging by CDFI of thrombus before and after treatment. **(A–D)** Corresponded to the Group I-IV before treatment. **(E–H)** Corresponded to the Group I-IV after treatment. The white arrow indicated the thrombus in the blood vessel.

Before treatment, solid hypoechoic thrombus was in the veins of the thrombosed segment (marked by the white arrow), and CDFI showed that there was no obvious blood flow signal in the veins in Group I-IV (Figures 2A–D). After treatment, there was still solid hypoechoic thrombus in the vein (marked by the white arrow). CDFI showed that there was a little discontinuous color blood flow signal around the thrombus in Group I (Figure 2E). Part of the vein was solid hypoechoic (marked by the white arrow) and the other part recanalized and enabled the accelerated color flow signal to pass through in Group II (Figure 2F). There was almost no thrombus in the vein of Group III, and continuous color blood flow signal passed through the vessel (Figure 2G). The thrombus in Group IV was completely dissolved, the wall structure was clear, and patency of the blood flow signal was returned (Figure 2H).

#### 3.2.2 Evaluation of DVT by CEUS

**FIGURE 3:**
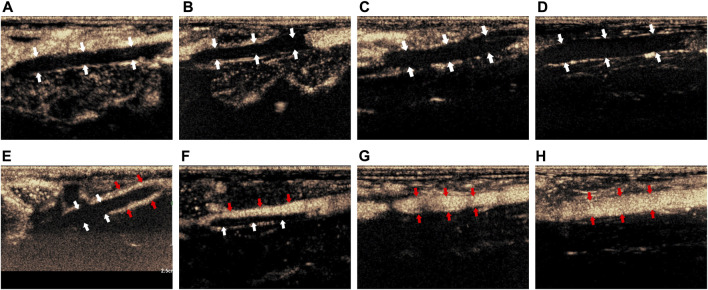
Comparison of imaging by CEUS of the thrombus before and after treatment. **(A–D)** Corresponded to the Group I-IV before treatment; **(E–H)** corresponded to the Group I-IV after treatment. The white arrow indicated the thrombus in the blood vessel, the red arrow indicated the contrast agent that passes through the vessel after recanalization.

Before treatment, CEUS showed that there was no obvious contrast agent in the veins of the thrombus segment of experimental animals in Group I-IV, but contrast agent could be seen in the surrounding tissue (Figures 3A–D). After treatment, CEUS showed that there was a very small amount of contrast agents around the thrombus in Group I (Figure 3E, marked by the red arrow). The contrast agent could pass through part of the vein (marked by the red arrow), while the other part was still blocked by the thrombus in Group II (Figure 3F). In Group III, the venous lumen was basically recanalized allowing the contrast agent to pass through (Figure 3G, marked by the red arrow). The vein was totally unobstructed, and the contrast agent passed through the blood vessel smoothly in Group IV (Figure 3H, marked by the red arrow).

The above results proved that before treatment, there was thrombus in the inferior vena cava of rabbits in Group I-IV. After treatment, there was little thrombolysis and recanalization of blood vessel in Group I; there was partial thrombolysis in Group II; the thrombus in Group III was almost dissolved; and the blood vessel in Group IV were entirely recanalized and the blood flow was unobstructed. This indicated that the treatment methods in Group I-IV were progressively effective.

### 3.3 The D-dimer content in each group

The comparison of D-dimer content in Group I-IV showed that, after treatment, the D-dimer content of rabbits in Group II-IV was lower than that in Group I (*p* < 0.05). However, there was no significant difference in D-dimer content among Group I-IV before modeling and treatment (*p* > 0.05) Table 1.

**TABLE 1:** The D-dimer content in each group (ng/ml, ‾x ± s).

Group	Before modeling	Before treatment	After treatment
Group I	24.470 ± 3.4533	50.633 ± 6.820^▽^	47.383 ± 3.872^▽^
Group II	23.509 ± 3.882	47.552 ± 6.466^▽^	32.512 ± 4.373*^▽▼^
Group III	24.231 ± 4.476	46.892 ± 4.961^▽^	30.324±4.387*^▽▼^
Group IV	25.212 ± 4.227	46.613 ± 6.789^▽^	28.923 ± 4.866*^▼^

*p < 0.05 compared with Group I. ^▽^ *p* < 0.05 compared with Before modeling. ^▼^ *p* < 0.05 compared with Before treatment.

Pairwise comparison of D-dimer content before modeling, before treatment, and after treatment showed that, before treatment, the D-dimer content of rabbits in Group I-IV was higher than that before modeling (*p* < 0.05). After treatment, the D-dimer content of rabbits in Group I-III was higher than that before modeling (*p* < 0.05), and that of rabbits in Group II-IV was lower than that before treatment (*p* < 0.05). There was no statistical significance among other groups (*p* > 0.05).

### 3.4 The TNF-α content in each group

Comparison of TNF-α content in Group I-IV showed that, after treatment, the TNF-α content in Group I -III was lower than that in Group IV (*p* < 0.05), but before modeling and treatment, the TNF-α content of rabbits in Group I-IV was not statistically significant (*p* > 0.05) Table 2.

**TABLE 2:** The TNF-α content in each group (pg/mL, ‾x ± s).

Group	Before modeling	Before treatment	After treatment
Group I	12.662 ± 1.979	55.270 ± 7.771^▽^	67.082 ± 8.457*^▽▼^
Group II	11.908 ± 1.901	54.373 ± 7.942^▽^	63.169 ± 4.788*^▽▼^
Group III	12.366 ± 1.879	59.933 ± 8.572^▽^	65.437 ± 8.462*^▽▼^
Group IV	13.144 ± 1.837	55.603 ± 8.502^▽^	15.922 ± 4.369^▼^

*p < 0.05 compared with Group IV. ^▽^ *p* < 0.05 compared with Before modeling. ^▼^ *p* < 0.05 compared with Before treatment.

Pairwise comparison of TNF-α content before modeling, before treatment, and after treatment showed that, before treatment, the TNF-α content of rabbits in Group I-IV was higher than that before modeling (*p* < 0.05). After treatment, the TNF-α content of rabbits in Group I-III was higher than that before modeling and treatment (*p* < 0.05); the TNF-α content of rabbits in Group IV was lower than that before treatment (*p* < 0.05), and there was no significant difference among other groups (*p* > 0.05). It showed that only the level of inflammatory factors in Group IV decreased, and the other groups had no significant effect on the inflammatory response.

### 3.5 Results of four indexes of coagulation in peripheral blood of rabbits

#### 3.5.1 PT in each group

Comparison of PT Group I-IV showed that, after treatment, PT of rabbits in Group II-IV was longer than that of Group I (*p* < 0.05); before modeling and treatment, PT of rabbits in Group I-IV was not statistically significant (*p* > 0.05). It showed that the use of UK could prolong PT, but the use of contrast medium did not affect PT (Table 3).

**TABLE 3:** PT in each (s, ‾x ± s).

Group	Before modeling	Before treatment	After treatment
Group I	7.098 ± 0.592	7.488 ± 0.603	7.479 ± 0.690
Group II	7.022 ± 0.601	7.351 ± 0.547	8.084 ± 0.447*^▽^
Group III	7.244 ± 0.476	7.532 ± 0.610	7.962 ± 0.362*^▽^
Group IV	7.303 ± 0.521	7.704 ± 0.687	8.011 ± 0.356*^▽^

*p < 0.05 compared with Group I. ^▽^ *p* < 0.05 compared with Before modeling.

Pairwise comparison of PT before modeling, before treatment, and after treatment showed that, after treatment, PT of rabbits in Group II-IV was higher than that before modeling (*p* < 0.05), and there was no statistical significance among other groups (*p* > 0.05).

#### 3.5.2 APTT in each group

Comparison of APTT in Group I-IV showed that, after treatment, the APTT of rabbits in Group II-IV was longer than that in Group I (*p* < 0.05), while before modeling and treatment, the APTT of rabbits in Group I-IV was not statistically significant (*p* > 0.05). It showed that the use of UK could prolong APTT, but the use of contrast medium did not affect APTT (Table 4).

**TABLE 4:** APTT in each group (s, ‾x ± s).

Group	Before modeling	Before treatment	After treatment
Group I	13.822 ± 1.419	13.204 ± 1.002	13.588 ± 1.441
Group II	13.593 ± 1.046	12.967 ± 1.426	16.304 ± 2.433*^▽▼^
Group III	14.023 ± 0.611	13.844 ± 1.512	16.408 ± 2.130*^▽▼^
Group IV	13.851 ± 0.773	13.710 ± 1.328	16.878 ± 2.010*^▽▼^

*p < 0.05 compared with Group I. ^▽^ *p* < 0.05 compared with Before modeling. ^▼^ *p* < 0.05 compared with Before treatment.

Pairwise comparison of APTT of rabbits before modeling, before treatment, and after treatment showed that, after treatment, APTT of rabbits in Group II-IV was higher than that before modeling and before treatment (*p* < 0.05), and there was no statistical significance among other groups (*p* > 0.05).

#### 3.5.3 TT in each group

Comparison of TT in Group I-IV showed that, after treatment, the TT of rabbits in Group II-IV was longer than that of Group I (*p* < 0.05), while before modeling and treatment, the TT of rabbits in Group I-IV was not statistically significant (*p* > 0.05). It showed that the use of UK could prolong TT, but the use of contrast medium did not affect TT (Table 5).

**TABLE 5:** TT in each group (s, ‾x ± s).

Group	Before modeling	Before treatment	After treatment
Group I	27.664 ± 5.261	26.039 ± 3.167	26.832 ± 4.209
Group II	28.211 ± 4.376	27.050 ± 3.447	35.739 ± 5.550*^▽▼^
Group III	28.878 ± 5.540	28.111 ± 2.624	32.986 ± 4.437*^▽▼^
Group IV	30.843 ± 3.631	28.681 ± 1.902	35.102 ± 4.513*^▽▼^

*p < 0.05 compared with Group I. ^▽^ *p* < 0.05 compared with Before modeling. ^▼^ *p* < 0.05 compared with Before treatment.

Pairwise comparison of TT before modeling, before treatment, and after treatment showed that, after treatment, the TT of rabbits in Group II-IV was higher than that before modeling and before treatment (*p* < 0.05), and there was no statistical significance among other groups (*p* > 0.05).

#### 3.5.4 The FIB content in each group

Comparison of FIB content in Group I-IV showed that, after treatment, the FIB content of rabbits in Group II-IV was lower than that in Group I (*p* < 0.05), but before modeling and treatment, the FIB content of rabbits in Group I-IV was not statistically significant (*p* > 0.05) (Table 6).

**TABLE 6:** The FIB content in each group (g/L, ‾x ± s).

Group	Before modeling	Before treatment	After treatment
Group I	1.087 ± 0.190	2.102 ± 0.289^▽^	1.944 ± 0.331^▽^
Group II	1.122 ± 0.231	1.882 ± 0.337^▽^	1.272 ± 0.241*^▼^
Group III	1.154 ± 0.227	1.971 ± 0.268^▽^	1.104 ± 0.223*^▼^
Group IV	1.202 ± 0.279	1.983 ± 0.330^▽^	1.027 ± 0.276*^▼^

*p < 0.05 compared with Group I. ^▽^ *p* < 0.05 compared with Before modeling. ^▼^ *p* < 0.05 compared with Before treatment.

Pairwise comparison of FIB content before modeling before treatment and after treatment showed that, before treatment, the FIB content of rabbits in Group I-IV was higher than that before modeling (*p* < 0.05). After treatment, the FIB content of rabbits in Group I was higher than that before modeling (*p* < 0.05), the FIB content of rabbits in Group II-IV was less than that before treatment (*p* < 0.05), and there was no statistical significance among other groups (*p* > 0.05).

### 3.6 Hematoxylin-eosin staining of thrombus

**FIGURE 4:**
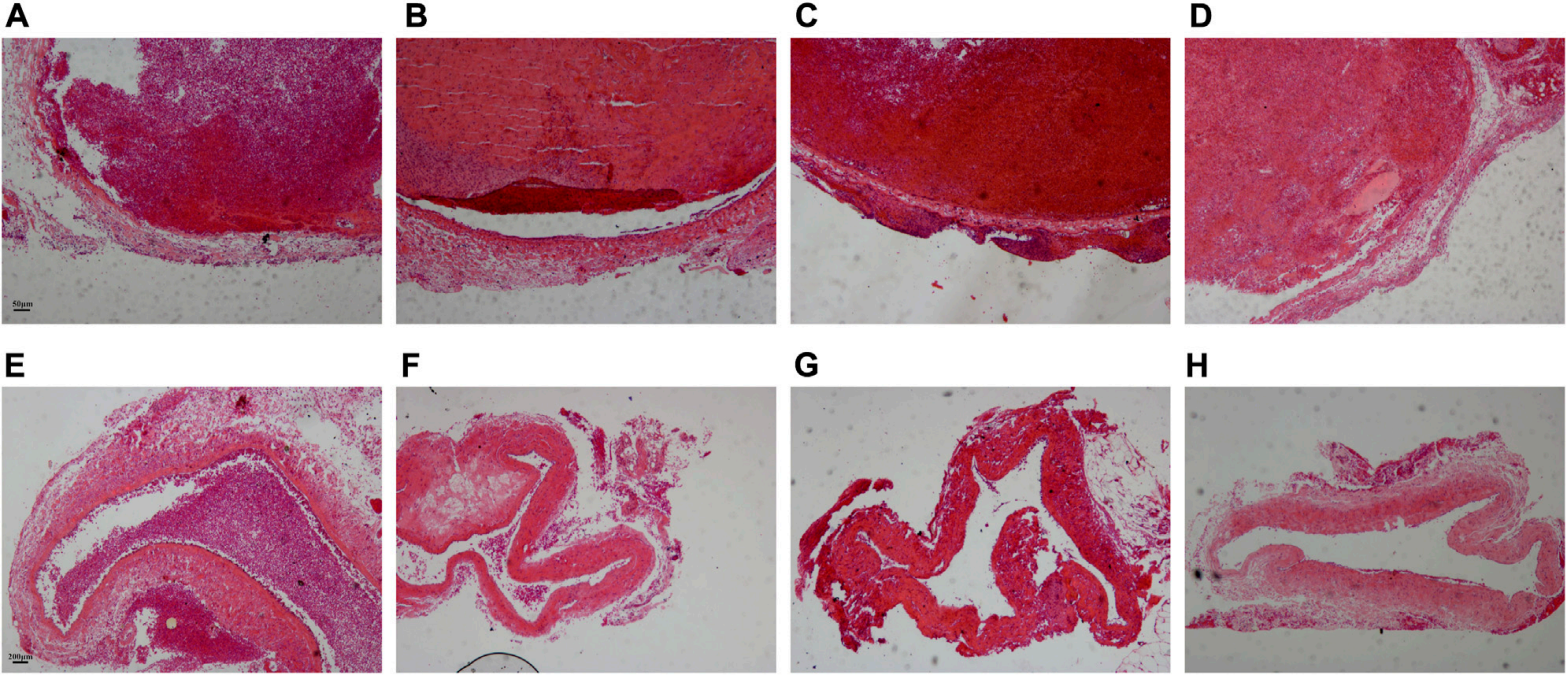
Comparison of pathological results of the thrombus before and after treatment. **(A–D)** Corresponded to the Group I-IV before treatment. Scale bar = 50 μm. **(E–H)** Corresponded to the Group I-IV after treatment. Scale bar = 200 μm.

Before treatment, the thrombus in the vascular lumen was uniform in texture and consisted of a large amount of red-stained cellulose interwoven in a network to form a fibrous mesh, which was filled with dense red blood cells. The boundary between the thrombus and vessel wall was clear, and there was no significant difference in the thrombus among Group I-IV (Figures 4A–D).

After treatment, the thrombus was reduced in all four experimental groups, and the density of red blood cells in the thrombus decreased. Only a small thrombus was dissolved in Group I, and the thrombus structure was looser than that before treatment, especially around the thrombus (Figure 4E). Most of the thrombi were dissolved except for a tiny remnant on one side of the blood vessel in Group II (Figure 4F). The thrombus was basically dissolved and the vessel wall structure was clear in Group III (Figure 4G). The thrombus was completely dissolved in Group IV and there was no obvious thrombus in the vein (Figure 4H).

## 4 Discussion

The application of ultrasound in thrombus therapy was first noted in 1976 (Trübestein et al., 1976). The pivotal mechanism is that the cavitation effect produced by ultrasonic irradiation promotes thrombolysis. Sonic flow generated by low-frequency ultrasound causes mechanical displacement of the thrombus and exposes the fiber structure inside the thrombus to thrombolytic medicine. The micro jet caused by the cavitation effect generates a shear force on the surface of the thrombus and then dissolves the thrombus (Pan et al., 2023).

In recent years, research on the application of ultrasound targeted contrast agents has improved rapidly (Liu et al., 2022c). CEUS is a highly secure inspection technology developed in recent years. The principle of this method is that ultrasound contrast agents reflect ultrasound and create different echo signals for the diagnosis of related diseases. MBt contrast agents are not only used for the diagnosis of diseases, but also carry genes or medicine to treat various diseases (Han et al., 2022). MBt swiftly expands and ruptures under the action of low-power focused ultrasound, then releases the genes or medication, so as to increase the local medicine concentration and enhance the efficacy without systemic side effects (Yu et al., 2020; Liu et al., 2023).

Many studies have verified that there is a close connection between inflammation and venous thrombosis (Weitz and Chan, 2020; Stark and Massberg, 2021). TNF-α is one of the critical factors in many diseases complicated with venous thrombosis (Zheng et al., 2021). It plays a vital role in mediating inflammatory events related to venous thrombosis, including early inflammatory response and neutrophil infiltration into the venous wall. Previous studies confirmed that the TNF-α content continued to rise after thrombosis. And the MBt contrast agent carrying monoclonal antibodies against IL-6, IL-8, and TNF-α was successfully prepared. Therefore, this research followed the preceding preparation method of MBt contrast agent, prepared MBt carrying TNF-α antibody, and selected TNF-α as the target to study the treatment of DVT.

This study concluded that there was no significant difference in PT, APTT, and TT of rabbits in each group before modeling and treatment, but the contents of FIB, D-dimer, and TNF-α increased significantly before treatment. Although relevant literature (Heestermans et al., 2021) reported that Pt and APTT would be shortened after thrombosis, the four indexes of coagulation were affected by numerous factors. Pt and APTT were not the most sensitive indicators to predict thrombosis. The relationship between thrombosis and the fibrinolytic system was complicated. In addition, this experimental model was an acute phase thrombus model. Therefore, these two indicators did not change significantly in this research after thrombosis. As the most sensitive index for clinical prediction of thrombosis, the contents of D-dimer and FIB increased markedly after thrombosis.

After treatment with different schemes, the four coagulation indexes and D-dimer content in Group I did not change significantly compared with that before treatment. But the content of TNF-α increased compared with those before treatment, indicating that ultrasonic irradiation did not increase the risk of bleeding or activate the fibrinolytic system *in vivo*, and had no effect on inflammatory reactions. The PT, APTT, and TT in Group II-IV were longer than those before and after modeling, while the contents of FIB and D-dimer were significantly lower than those before modeling and before treatment, indicating that the use of UK to treat thrombosis could also increase the risk of bleeding. Among the four groups, only the TNF-α content in Group IV decreased significantly after treatment, indicating that TNF-α content increased with the extension of thrombosis time, and MBt effectively reduced the level of the inflammatory factors.

Meanwhile, there was no significant difference in the four indexes of coagulation and the contents of D-dimer and TNF-α among the rabbits in Group I-IV before modeling and before treatment. After treatment, the PT, APTT, and TT of rabbits in Group II-IV were longer than those in Group I, and the contents of FIB and D-dimer of rabbits in Group II-IV were lower than those in Group I; in addition, there was no significant difference in the four indexes of coagulation and the content of D-dimer among Group II-IV. It showed that both MBc and MBt would not cause changes in the coagulation system. As for the content of TNF-α, Group IV was higher than Group I-III, but there was no significant difference among Group I-III. It showed that in thrombotic diseases, the increase of inflammatory factor level was not only related to thrombosis, but may also be linked to reperfusion injury after thrombolysis.

Through the analysis of CDFI, CEUS, and pathological results, the study found that the thrombolysis of focused ultrasound alone was limited. When focused ultrasound was combined with UK, the thrombus was partially dissolved. With the addition of a microbubble contrast agent, the thrombus almost disappeared, which indicated that the treatment method significantly accelerated the thrombolysis and promoted the recanalization of blood vessels. Furthermore, the combination of UK, focused ultrasound, and MBt produced the best therapeutic effect. More MBt accumulates locally in the thrombus through an antigen-antibody reaction, facilitating the therapeutic effect of UK on the thrombus, so as to completely dissolve the thrombus while alleviating the inflammatory response.

Combined with previous studies, we found that CEUS can be used not only to diagnose thrombosis but also to treat it. The thrombolytic effect of ultrasound was one of its clinical applications, which mainly depended on the cavitation effect to enhance the efficacy of thrombolytic medicine (Porter et al., 2017; Ma et al., 2020). Relevant studies showed that a microbubble contrast agent, as an artificially added cavitation core, could enhance the induced ultrasonic cavitation effect and improve the thrombolytic effect of ultrasound (Yang and Zhou, 2017).

In summary, this study had some innovations for thrombosis treatment. At first, the combination of targeted microbubbles and UK was innovatively used in the study of acute DVT. Thrombus was diagnosed by ultrasound imaging and treated at the same time. This treatment has been rarely reported. Secondly, TNF-α was used as the target of thrombus to attract a large number of targeted microbubbles to enrich locally in the thrombus and neutralize the inflammatory response, which was another innovative way to treat thrombus. This experiment opens up new ways to study the effect of UTMD technology targeting inflammatory factors on thrombosis and provides new ideas for the diagnosis and treatment of acute DVT.

This study has a few limitations in experimental design. This experiment only treated acute thrombosis, ignoring the long-term damage of thrombus. By extending the experimental time, we will prepare animal models of thrombosis in the subacute and chronic phases and conduct therapeutic studies on them. Secondly, using low-power focused ultrasound to thrombi could affect the perfusion of microcirculation of the surrounding tissues. The main concern in the experiment was the recanalization of vascular lumen. We will study the damage to the vessel wall of the thrombus segment and use more refined ultrasound irradiation to avoid damage to the tissue surrounding the thrombus.

## Data Availability

The original contributions presented in the study are included in the article/Supplementary Material, further inquiries can be directed to the corresponding author.
